# Predictors of binge drinking in adolescents: ultimate and distal factors - a representative study

**DOI:** 10.1186/1471-2458-12-263

**Published:** 2012-04-02

**Authors:** Carolin Donath, Elmar Gräßel, Dirk Baier, Christian Pfeiffer, Stefan Bleich, Thomas Hillemacher

**Affiliations:** 1 Department Medical Psychology and Medical Sociology, Psychiatric University Hospital Erlangen (Friedrich-Alexander-Universität Erlangen-Nürnberg), Schwabachanlage 6, 91054 Erlangen, Germany; 2 Criminological Research Institute of Lower Saxony, Lützerodestr. 9, 30161 Hannover, Germany; 3 Center for Addiction Research, Clinic for Psychiatry, Social Psychiatry and Psychotherapy, Hannover Medical School, Carl-Neuberg-Str. 1, 30625, Hannover, Germany

## Abstract

**Background:**

As epidemiological surveys have shown, binge drinking is a constant and wide-spread problem behavior in adolescents. It is not rare to find that more than half of all adolescents engage in this behavior when assessing only the last 4 weeks of time independent of the urbanity of the region they live in. There have been several reviews on predictors of substance consumption in adolescents in general, but there has been less high quality research on predictors of binge drinking, and most studies have not been theoretically based. The current study aimed to analyze the ultimate and distal factors predicting substance consumption according to Petraitis' theory of triadic influence. We assessed the predictive value of these factors with respect to binge drinking in German adolescents, including the identification of influence direction.

**Methods:**

In the years 2007/2008, a representative written survey of N = 44,610 students in the 9^th ^grade of different school types in Germany was carried out (net sample). The return rate of questionnaires was 88% regarding all students whose teachers or school directors had agreed to participate in the study. In this survey, prevalence of binge drinking was investigated as well as potential predictors from the social/interpersonal, the attitudinal/environmental, and the intrapersonal fields (3 factors of Petraitis). In a multivariate logistic regression analysis, these variables were included after testing for multicollinearity in order to assess their ability to predict binge drinking.

**Results:**

Prevalence of binge drinking in the last 30 days was 52.3% for the surveyed adolescents with a higher prevalence for boys (56.9%) than for girls (47.5%). The two most influential factors found to protect against binge drinking with *p *< .001 were low economic status and importance of religion. The four most relevant risk factors for binge drinking (*p *< .001) were life-time prevalence of school absenteeism/truancy, academic failure, suicidal thoughts, and violence at school in the form of aggressive behavior of teachers. The model of Petraitis was partly confirmed for Binge Drinking in German adolescents and the direction of influence factors was clarified.

**Conclusions:**

Whereas some of the risk and protective factors for binge drinking are not surprising since they are known for substance abuse in general, there are two points that could be targeted in interventions that do not focus on adolescents alone: (a) training teachers in positive, reassuring behavior and constructive criticism and (b) a focus on high risk adolescents either because they have a lack of coping strategies when in a negative mood or because of their low academic achievement in combination with absenteeism from school.

## Background

Problematic alcohol consumption patterns - including binge drinking - are constant evident behaviors in many adolescents across Europe and the USA [1-3] with some differences according to the migration background of an adolescent and according to urban or rural residence [4,5]. Aside from the direct consequences of intoxication [6] and its possible somatic complications, the long-term consequences of this consumption pattern are disadvantages in different social areas of life (school, education, job perspectives; risky behavior in traffic and sexual activity [7,8]; delinquency [9]) and according to the latest research, also biological changes in neuronal processes of the hippocampus, likely resulting in memory and cognitive deficits [10] and altered emotional competence [11].

The 2007 ESPAD report (European survey of 15-/16-year-olds concerning substance use in 35 European countries) states that heavy episodic drinking (having had 5 or more drinks on one occasion in the last 30 days) varies across Europe between 20% (Iceland) and 60% (Denmark). No data are reported for Germany. Except for the north-western part of Europe, boys more often consume heavily on any one occasion than girls [12,13]. The German Federal Center for Health Education regularly carries out a representative survey of 12-to-17-year-olds concerning their substance consumption. The 2008 data concerning alcohol consumption show that alcohol was the most widely used psychoactive substance: three-fourths of the adolescents stated that they use it. 17.4% of the adolescents consume alcohol weekly or more often, again, boys in a greater proportion than girls [14]. Binge drinking (same definition as in the ESPAD study) is reported by 20.4% of the 12-to-17-year-olds. Both are representative studies. A review investigating binge drinking epidemiology in the UK across a time period of 25 years concentrating on university students shows a high variety of binge drinking (partly due to the different definitions of binge drinking and sex differences) between 24% and 64% [15]. For the United States, it is reported in a Review by Courtney and Polich [5] that 19% of all adolescents between 12 and 20 years engaged in binge drinking in the last 4 weeks (same definition as [12-14]).

There have been several reviews exploring the predictors - meaning risk factors as well as protective factors - of substance consumption in adolescents mostly based on studies from the North American context [e.g. [16,17]] and several more single studies on this theme. There are fewer reviews on predictors of binge drinking - a special risky consumption behavior - in the international context. However, whereas the existing reviews have begun to assess predictors of binge drinking, these assessments were either not based on a theoretical framework [e.g. [3,5]] or they concentrated only on a certain group of predictors such as alcohol expectancies and drinking refusal self-efficacy [e.g. [18]].

The current study aimed to analyze predictors of binge drinking based on a theoretical framework and concentrated on those predictors not immediately connected to the consumption behavior itself. The model chosen was the theory of triadic influence developed by Petraitis et al. [16] who arranged predictors of "illicit substance use" in a matrix of nine fields (see Table 1). There are ultimate, distal, and proximal influence factors, and each of those can be divided into social, attitudinal, and intrapersonal risk/protective factors. Additionally, Petraitis and colleagues [16] mention trial behavior and intentions as immediate influence factors.

**Table 1:** Matrix of influence factors of illicit substance use (ISU) according to Petraitis et al. (1998)

Level of influence	Types of influence		
	
	Social/Interpersonal	Attitudinal/Environment	Intrapersonal
Ultimate	*Definition*: Characteristics of the people who make up adolescents' most intimate social support system. These characteristics are not specific to ISU and are beyond the personal control of adolescents *Constructs*: Infrequent opportunities for rewards from family members; lack of parental warmth, support, or supervision; negative evaluations from parents; home strain; parental divorce or separation; unconventional values of parents; unconventional values among peers	*Definition*: Aspects of adolescents' immediate surroundings, neighborhoods, social institutions, and culture that, although beyond the personal control of adolescents, put them at risk *Constructs*: Local crime and employment rates; inadequate schools; poor career and academic options; infrequent opportunities for rewards at school; negative evaluations from teachers; media descriptions of ISU; availability of substances; weak public policies on ISU	*Definition*: Personality traits and intrapersonal characteristics that, although beyond the easy control of adolescents, might promote some internal motivation or make them susceptible to the physiological effects of ISU. *Constructs*: Genetic susceptibility to addiction; lack of impulse control; external locus of control; aggressiveness; extroversion; sociability; risk-taking; sensation-seeking; neuroticism or emotional instability; intelligence

Distal	*Definition*: Emotional attachments of adolescents and the substance-specific attitudes and behaviors of influential role models who encourage ISU *Constructs*: Weak attachment to and weak desire to please family members; strong attachment to and strong desire to please peers; greater influence of peers than parents; substance-specific behaviors of role models	*Definition*: Personal values and behaviors of adolescents that contribute to their attitudes toward ISU *Constructs*: Weak commitment to conventional values, school, and religion; social alienation and criticism; weak desire for success and achievement; hedonic values and short-term gratification; rebelliousness; desire for independence from parents; tolerance of deviance	*Definition*: Affective states and general behavioral skills of adolescents that promote some internal motivation for ISU and that undermine their refusal skills *Constructs*: Low self-esteem; temporary anxiety, stress, or depressed mood; poor coping skills; inadequate social skills; weak academic skills

Proximal	*Definition*: Beliefs about the normative nature of ISU and pressures to use substances *Constructs*: Prevalence estimates; motivation to comply with other users; beliefs that important others (i.e., friends, parents, and other role models) encourage ISU	*Definition*: Beliefs and evaluations about the costs and benefits of ISU *Constructs*: Expected costs and benefits of ISU; evaluation of costs and benefits of ISU; attitudes of others toward ISU; attitudes of self toward ISU	*Definition*: Beliefs about one's ability to use or to avoid substances *Constructs*: Refusal skills; determination to use substances; use self-efficacy; refusal self-efficacy

Immediate		Decision/intentions; trial and related behavior	

Many existing prevention strategies focus on factors that are relatively "close" to the consumption behavior (i.e., proximal factors). These prevention strategies try to influence these proximal factors (e.g., training refusal skills, developing refusal-skill efficacy) to thus prevent harmful consumption behavior (e.g., the DARE program in the USA [19]). However, it is known that effective prevention strategies target several life areas ("mesosystems" in the sense of Bronfenbrenner) such as family, school, community, society (legislation), and the person him- or herself, and include also more distal and ultimate factors to substance consumption [20].

The aim of this study was to analyze the predictive value of ultimate and distal influence factors for binge drinking based on a representative study of German adolescents and based on the theoretical predictor framework (theory of triadic influence) of Petraitis [16]. Within this framework, the goal was to clarify the direction of influence in the sense of risk and protective factors. With the results, it will hopefully be possible to develop effective prevention measures for binge drinking. The added knowledge for the scientific community includes: a) applying the theoretical model to binge drinking and b) examining whether the existing knowledge accounts for Germany as well.

### Aims

Analysis of ultimate and distal predictors of binge drinking in a representative sample of German adolescents

## Methods

### Design

The matter concerns a representative survey of 9^th ^graders in Germany conducted in 2007/2008. In the year 2006, there were 910,000 9^th ^graders in Germany. The goal was to survey 50,000 adolescents from different regions. The basis for the selection of the regions was the federal classification of rural districts and independent cities (urban districts), which total 440. The urban districts contain cities of each size (below 100,000 and up to 3.3 million (Berlin) inhabitants). The number of inhabitants in the rural districts also varies from about 50,000 to over 600,000. Therefore, the rural and urban districts (i.e., regions) were sorted into classes of region size from which the random drawing then took place. The classes of region size were: Western Germany (urban districts): cities with more than 500,000 inhabitants, cities with more than 100,000 inhabitants, cities with fewer than 100,000 inhabitants; Western Germany (rural districts): districts with more than 100,000 inhabitants, districts with fewer than 100,000 inhabitants; Eastern Germany (former GDR) (urban districts): cities with more than 100,000 inhabitants (there are only two cities with more than 500,000 inhabitants), cities with fewer than 100,000 inhabitants; Eastern Germany (former GDR) (rural districts): districts with more than 100,000 inhabitants, districts with fewer than 100,000 inhabitants; special case: Berlin. With the knowledge about the number of 9^th ^graders in each class of region size (from the official education statistics) and the goal of 50,000 adolescents to be questioned, it was possible to calculate how many adolescents per class of region size had to be included. Note that classes were drawn by chance, but students were not. The number of 50,000 students refers to a goal of 2,500 classes. It was known that about 20 students per class could be retrieved and used for data analysis. The number of 2,500 classes was chosen in such a way that for every region in Germany that was supposed to be represented in the survey, a sufficient number of classes was evident. The goal was to match the distribution of the 9^th ^graders in the classes of region size (in the population) to the same percentage in the sample. It was assumed that every 2^nd ^student (in large cities, every 6^th ^student) in a drawn region would be questioned. Thus, we could calculate how many regions had to be drawn out of every class of region size. These steps resulted in 61 regions. Which region was chosen to take part was then drawn by chance in order to secure a representative sample. At the Criminological Research Institute of Lower Saxony, the sample was drawn stratified by school type (on the basis of school lists provided by the local education authorities). A master list in which all school classes (9^th ^grade) of one region were consecutively sorted was used. Then all directors of the drawn schools were informed in writing about the survey and asked for the participation of their 9^th^-grade school classes. If the directors agreed to the survey, information material including consent forms for parents were sent to the schools. On an appointed day, the written survey was administered without the students whose parents refused participation, who themselves refused to participate, or who were otherwise busy or absent during the survey. The survey at the school was carried out by trained external study assistants - not by the employees of the schools - in order to preserve reliability and validity.

The research project was granted by the Federal Ministry of the Interior in Germany; thus, a statement of an ethics committee was not necessary. Instead, the survey was audited by each Ministry of Education of every German state (Bundesland) and additionally of every state responsible for data protection. The survey then actually took place only in those states in which the survey was permitted after following this procedure. A further ethics committee was not included since the data protection matters were covered by the above described procedure and no other intervention besides filling out an anonymous questionnaire was applied. One manuscript based on this data set has already been published. That study concerns epidemiological data on binge drinking according to differences in urban and rural areas and concerning migration background [4].

### Instruments

The item assessing heavy episodic drinking (binge drinking) was derived from the representative survey of adolescents of the German Federal Center for Health Education [14]. Binge drinking is defined as the consumption of five or more standard drinks at one drinking opportunity. The adolescents were asked a) if they had consumed alcohol in the last 30 days (30-day-prevalence) and if yes, b) on how many days they had consumed 5 or more standard alcoholic drinks in a row. The answer categories were a) yes/no and b) not on one day, on one day, on two days, (...), on 20 or more days.

The following paragraphs describe the variables that were chosen to operationalize the constructs of Petraitis et al. [16] (see Table 2).

**Table 2:** Operationalization of the ultimate and distal potential influence factors of binge drinking

	Constructs: Social/Interpersonal	Operationalization	Constructs: Attitudinal/Environment	Operationalization	Constructs: Intrapersonal	Operationalization
Ultimate	- Infrequent opportunities for rewards from family members - Lack of parental warmth, support, or supervision - Negative evaluations from parents - Home strain - Parental divorce or separation - Unconventional values of parents - Unconventional values among peers	- Acknowledgment of success/rewards by parents - Parental warmth in childhood - Parental control/supervision in adolescence - Parental separation events - Cultural communication in the family	- Local crime and employment rates - Inadequate schools - Poor career and academic options - Infrequent opportunities for rewards at school - Negative evaluations from teachers - Media descriptions of ISU - Availability of substances - Weak public policies on ISU	- Community/neighborhood cohesion - Community/neighborhood/school safety - Welfare status - Violence level in the school - Willingness of teachers to intervene during violent conflicts - Violence/problems at school - aggressive behavior of teachers	- Genetic susceptibility to addiction - Lack of impulse control - External locus of control - Aggressiveness - Extroversion - Sociability - Risk-taking - Sensation-seeking - Neuroticism or emotional instability - Intelligence	- Attention deficit disorder - Risk-taking behavior - School grades

Distal	- Weak attachment, weak desire to please family members - Strong attachment, strong desire to please peers - Greater influence of peers than parents - Substance-specific behaviors of role models	- Number of friends - Number of delinquent friends - Deviant/Assimilated behavior of one's own group of friends - Smoking parents	- Weak commitment to conventional values, school, and religion - Social alienation and criticism - Weak desire for success and achievement - Hedonic values and short-term gratification - Rebelliousness - Desire for independence from parents - Tolerance of deviance	- Voluntary non-profit activities - Religiosity - School commitment - Social integration in school - Social Desirability/Conventional Values - Planned type of school leaving certificate - Absenteeism/Truancy - Hedonistic reasons for truancy	- Low self-esteem - Temporary anxiety, stress, or depressed mood - Poor coping skills - Inadequate social skills - Weak academic skills	- Self-esteem - Mental well-being/mood - School anxiety - Suicidal thoughts - Mandatory repetition of school year

The following potential predictors were measured:

1. Acknowledgment of success/rewards by parents: The students were asked whether they achieved something to be proud of in the last 12 months in the areas sports, music, friends, family, school, computer games, society, or work and from whom they received the acknowledgment. A sum score of fatherly and motherly acknowledgment across the eight areas was built. The items were constructed by the Criminological Research Institute of Lower Saxony.

2. Parental warmth in childhood: A scale based on the concept of parental style by Baumrind [21] (translated by Wilmers et al. [22]) was used. It consists of six items exploring parental warmth in childhood for mother and father separately. Cronbach's alphas were .86 (motherly warmth) and .90 (fatherly warmth). A sum score was used for parental warmth.

3. Parental control/supervision in adolescence: A scale based on the concept of parental style by Baumrind [21] (translated by Wilmers et al. [22]) was used. It consists of three items exploring parental control and supervision in adolescence in the last 12 months for mother and father separately. Cronbach's alphas were .76 (motherly control) and .80 (fatherly control). A sum score was used for parental control.

4. Parental separation events: The students were asked whether their parents were separated or divorced or whether their mother or father had died. If one of the items was answered yes, the student received a 'positive' parental separation score. The items were constructed by the Criminological Research Institute of Lower Saxony.

5. Cultural communication in the family: A scale of two items developed by Kunter et al. [23] based on the theory of cultural capital by Bourdieu [24] was used. These items explore whether it is usual for the student's family to talk about political or social questions and whether it is usual to talk about books, movies, or TV broadcasts. Cronbach's alpha of the scale was .77. The sum score of the two items was used.

6. Number of friends: The students were asked about the number of friends with whom they spend time outside of school. The item was constructed by the Criminological Research Institute of Lower Saxony.

7. Number of delinquent friends: The number of friends known by the student with at least one delinquent behavior in the last 12 months was assessed. Five different delinquent behaviors were listed (e.g., selling illicit drugs). A sum score of delinquent friends was built. The item was constructed by the Criminological Research Institute of Lower Saxony.

8. Deviant/Assimilated behavior of one's own group of friends: Different delinquent behaviors in the group of friends of the student, including dealing drugs, were assessed with five items, and socially acceptable adolescent group behaviors that don't break laws were assessed with two items. The items were formulated in the "we"-perspective meaning the student is engaged actively or passively in the behavior himself. A sum score for deviant behavior and a sum score for assimilated behavior were used. The items were constructed by Wetzels et al. [25].

9. Smoking parents: We asked whether the student's mother or father regularly smokes. If one or both parents engage in smoking, the student received a 'positive' value on this variable.

10. Community/neighborhood cohesion: A scale developed by Sampson et al. [26] (translated by Oberwittler [27]), consisting of five items, was used. A sample item is "People in my neighborhood help each other." Cronbach's alpha of the scale was .78. The sum score was used for the analysis.

11. Community/neighborhood/school safety: A scale developed by Wilmers [22], consisting of five items, was used. A sample item is "How safe do you feel when you are at home in your apartment?" Cronbach's alpha of the scale was .75. The sum score was used for the analysis.

12. Welfare status: The students were asked whether their parents or they themselves lived on social welfare (unemployment pays "Hartz IV" welfare aid according to German social legislation). If they answered yes (versus no or I don't know) the student received a 'positive' welfare status score. The item was constructed by the Criminological Research Institute of Lower Saxony.

13. Violence level in the school: The construct was assessed with two items developed by Wilmers et al. [22] asking for violence in the school and fights and trouble among the students. A sum score of the two items was used.

14. Willingness of teachers to intervene during violent conflicts: The construct was assessed with two items developed by Olweus [28] asking whether teachers intervene when students fight violently and whether teachers prefer to look the other way if brawling among students occurs. A sum score of the two items was used.

15. Violence/problems at school - aggressive behavior of teachers: Three items describing verbal assaults and violent behavior of teachers against the students were used. The student was asked whether he had ever experienced one or more of those behaviors. If the student answered yes, he received a 'positive' score. The items were constructed by the Criminological Research Institute of Lower Saxony.

16. Non-profit volunteer activities: The students were asked for six different non-profit volunteer activities (e.g., working as a trainer for children) concerning their current involvement. An involvement score was built across the six areas. Past involvement was not counted. The item was constructed by the Criminological Research Institute of Lower Saxony.

17. Religiosity: The construct was assessed with a single item by Wetzels et al. [29]: "How important is religion for you personally?" which could be answered on a scale with five levels.

18. School commitment: Bonding to school was assessed with two items asking how much a student likes to go to school and how strongly he agrees with the statement that he really likes his school. A sum score of the two items was used. They were constructed by the Criminological Research Institute of Lower Saxony.

19. Social integration in school: The extent to which a student is integrated and accepted at school was assessed with two items asking for a self-rating of one's popularity with other students and the self-rated estimation of having lots of friends at school. Both items were rated on a 4-point graduated scale; a sum score of the two items was used. They were constructed by the Criminological Research Institute of Lower Saxony.

20. Social Desirability/Conventional Values: The construct was assessed with the revised version of the Social Desirability Scale by Crowne & Marlowe [30]. The German version of the scale was developed by Lück & Timaeus [31]. The scale consists of four items, with a 4-point graduated rating and is intended as a sum scale. However, reliability analysis in this sample showed an unsatisfying internal consistency with Cronbach's alpha = .20. A subsequent factor analysis showed that the four items loaded on two separate factors. Therefore, the four items were dichotomized, and an index with possible values from 0 to 4 was built. Higher values indicate higher social desirability.

21. Planned type of school leaving certificate: A single item with three answer categories was used to assess the planned type of school leaving certificate. According to the German school system, it was possible to choose between special school/secondary general school certificate (9 years) "Hauptschulabschluss," secondary modern school certificate (10 years) "Realschulabschluss," or general qualification for university entrance/hiqh school diploma "Abitur." The item was constructed by the Criminological Research Institute of Lower Saxony.

22. Absenteeism/Truancy: Students were asked to indicate whether the item "I have so far never been truant a whole day" was true for them. All students who did not check the item received a 'positive' truancy score. The item was constructed by Wilmers et al. [22].

23. Hedonistic reasons for truancy: Those students who admitted to having been absent without excuse (truancy) at least for one school lesson or one school day in the last half year were asked for the reasons. Two reasons displaying hedonistic attitudes were used for the analysis: "because I wanted to sleep in" and "because I was not in the mood for school." Students answering yes to one or both items received a 'positive' hedonistic values score. The items were constructed by Wilmers et al. [22].

24. Attention deficit disorder: The presence of an attention deficit disorder, which has high impulsivity as a diagnostic criterion, was asked with a single item developed by the Criminological Research Institute of Lower Saxony. The student had to answer whether a psychologist or a doctor had ever diagnosed an attention deficit disorder.

25. Risk-taking behavior: Risk-taking was assessed based on the concept of Grasmick et al. [32] with a four-item scale in a German translation by Wilmers et al. [22]. The internal consistency measured by Cronbach's alpha was satisfying (α = .85). A sum score across the four items was used for analysis.

26. School grades: A mean school grade was computed for the three self-stated school grades in Math, German, and History. The item assessing the school grades was constructed by the Criminological Research Institute of Lower Saxony.

27. Self-esteem: The construct was assessed with a scale developed by Ravens-Sieberer et al. [33] and is part of the KINDL questionnaire, which assesses health-related quality of life in children and adolescents with a total of six dimensions. The dimension self-esteem consists of four items showing a Cronbach's alpha of .61. A sum score across the four items was used for analysis. A sample item is: "In the last week, I was proud of myself."

28. Mental well-being/mood: The construct was assessed with a scale developed by Ravens-Sieberer et al. [33], and is also part of the KINDL questionnaire. The dimension mental well-being/mood consists of four items showing a Cronbach's alpha of .56. A sum score across the four items was used for analysis. The following is a sample item: "In the last week, I felt lonely."

29. School anxiety: The construct was assessed with a scale developed by Wilmers et al. [22] consisting of five items, for example: "I often cannot fall asleep because I am worried about school." The internal consistency measured with Cronbach's alpha was .79. A sum score across the five items was used in the analysis.

30. Suicidal thoughts: This aspect was assessed with a single item asking how often the student had already thought about suicide. The item was constructed by the Criminological Research Institute of Lower Saxony.

31. Mandatory repetition of school year: An item assessing a German specificity of the school system was included. It is possible that because of weak academic skills, a student is forced to repeat a whole school year. This aspect was assessed with a single item developed by the Criminological Research Institute of Lower Saxony: "Did you ever have to repeat a class?"

### Sample

**Figure 1:**
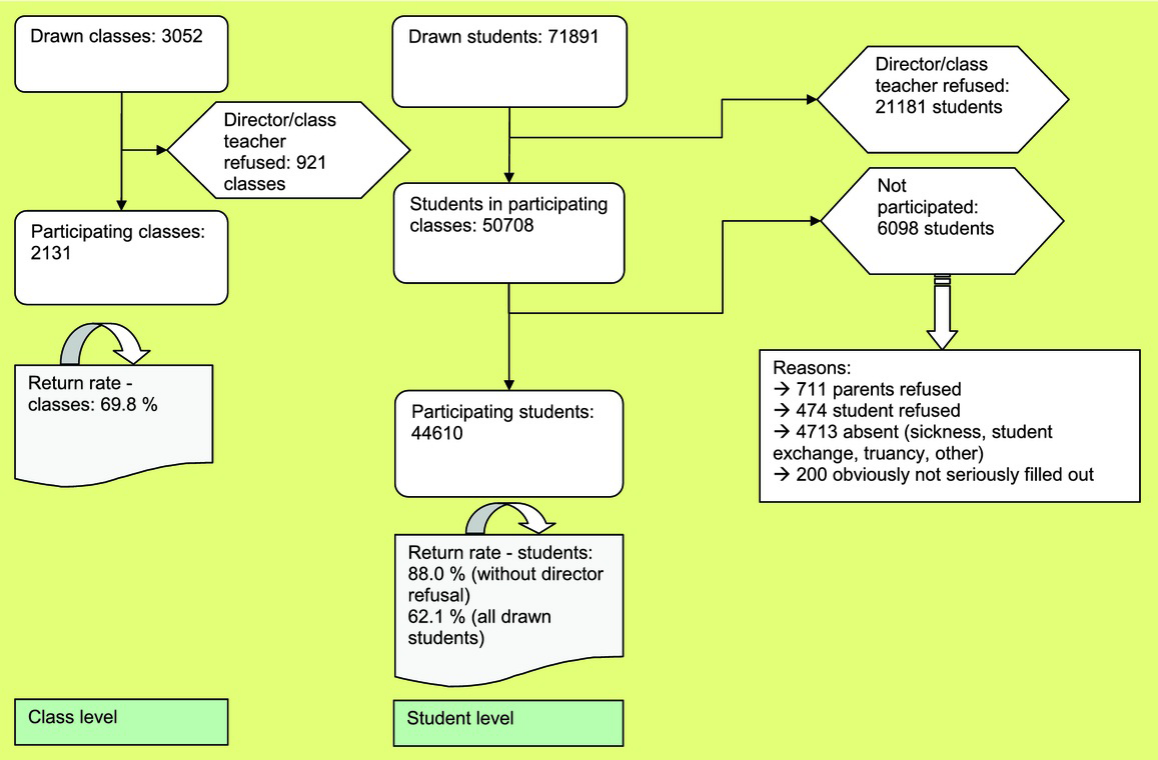
**Sample constitution**.

A total of 3,052 classes (9^th ^grade) were drawn. For 921 classes, the directors/main class teachers refused to participate. 2,131 classes participated. Actually, the 2,131 classes included 50,708 students, but 6,098 of them did not participate (reasons, for example: parents' refusal or absenteeism). Therefore the total sample size was 44,610 students (return rate 88%). Figure 1 comprises a detailed flow-chart of the sample record.

The return rates (students, without director refusal) differed between the school types in that grammar/secondary schools as well as private/not state-run schools had the highest return rates (92.0/92.8) and special schools the lowest (75.5). Furthermore, the return rates differed across the classes of region size. In the large cities, the return rate was lower in comparison with rural districts and urban districts with fewer than 500,000 inhabitants. In spite of the varying return rates in the different classes of region size, the final sample represented the proportions of the population very well (e.g., students living in cities with more than 100,000 inhabitants in Western Germany: 12.04% in the sample and 11.68% in the population). The proportion of students in the 9^th ^grade in every class of region size in Western and Eastern Germany was compared to their proportion in the sample. With those two percentages for each category, the reliability can be seen and rated. The proportions never differed more than 0.36% between population and sample in the different classes of region size except for Berlin where the difference was 0.62%.

To address the varying return rates, weighting factors were calculated so that the proportion of school forms in the sample corresponded to that in the population, and in the same manner, the proportion of regions with different sizes in the sample corresponded to that in the population. The two weighting factors were multiplicatively connected when the data from the total sample were analyzed. Thereby the imbalances regarding the school forms were eliminated, as were the much smaller imbalances regarding the classes of region size.

The sample can be characterized as follows: 51.3% of the sample was male, the mean age was 15.3 (SD 0.7) years. The percentage of adolescents with a migration background was 27.4%, whereby students with a Turkish migration background constituted the largest group (6.0%; more than 2,600 students) followed by emigrants from the former Soviet Union states (5.8%; more than 2,500 students). A total of 12.2% lived in large cities with more than 500,000 inhabitants including Berlin, whereas the majority lived in rural districts (68.8%). The migration background varied between 39.9% in large cities with more than 500,000 inhabitants and 23.9% in rural districts.

### Statistical analysis

After operationalization of the six defined variable groups by Petraitis, the resulting 31 variables were analyzed concerning multicollinearity. The goal was to get a lean but well operationalized model. We determined that variables with a medium (r > .5) or even high (r > .7) correlation with other variables needed to be reduced. Taking only the sample size into account, it would have been possible to include a large number of predictors. According to Altman [34], the number of independent variables used should not exceed the square root of the sample size (here n = 44,610; potential predictors > 200). However, we decided that a lean model was still a priority. Correlation coefficients were computed according to the measurement level of the variables, for example, Pearson's r for metric variables, Cramer's V (Phi) for categorical variables, etc. In consequence of the multicollinearity analysis, three pairs of variables showed a medium or high correlation: a) parental warmth in childhood with parental control/supervision in adolescence (r = .737). We decided to keep the variable parental warmth in childhood because more single items were used for the sum score of the scale than in the parental control construct and therefore variance in the variable parental warmth was higher. As a consequence, parental control in adolescence was not included as a predictor in the multivariate analysis. b) Absenteeism/Truancy with Hedonistic reasons for truancy (Phi = .516). Since hedonistic reasons for truancy is logically subordinate to absenteeism, and furthermore, hedonistic reasons were the most frequently named reasons for truancy, the item hedonistic reasons for truancy was omitted from the multivariate analysis and the item Absenteeism/Truancy was kept in the analysis. c) Number of delinquent friends with Deviant behavior in one's own group of friends (r = .601); The variable deviant behavior in one's own group of friends was kept in the analysis because the variable was comprised of a sum score built across several items, therefore having more variance than a single item like number of delinquent friends, which was omitted from the multivariate analysis.

The remaining variables were included as predictors in a multiple binary logistic regression analysis with binge drinking as the dependent variable. The independent variables were included in the regression equation by the enter method. As a measure for explained variance of the model, R^2 ^according to Nagelkerke, was used. Statistical analysis was performed with PASW 18.0. Because of the sample size, the level of significance was set to *p *< .001 [35];

however, statistical significance is not equivalent to clinical relevance, especially in large samples [36-38]. Therefore, Odds Ratios and their confidence intervals were also used for interpretation of results. Even though in general the use of Odds Ratios in comparison to other effect sizes has been discussed in the literature, it has been explicitly suggested that for logistic regressions [39], the interpretation of Odds Ratios in the sense of risk can be done safely when effect sizes are not large. In these cases, the interpretation of Odds Ratios is unlikely to lead to qualitatively different judgments about the study results [40]. Since the largest effects in our study showed a risk of increasing or decreasing below 40%, the over interpretation of effects by interpreting Odds Ratios in the sense of relative risks was relatively small according to the table in the publication of Davies et al. [40]. There is no published predefined level of Odds Ratio clearly indicating relevance of a predictor for all kinds of studies. Rather, clinical relevance has to be defined by experts, which in this case were the study authors [36-38]. We decided to interpret a predictor as clinically relevant in our study if the Odds Ratio was higher than 1.2 or smaller than 0.8 in combination with a *p*-value below .001. Predictors which change the risk to an OR of at least 1.1 resp. 0.9 at a *p*-level of < .001 are further interesting to consider being on the threshold to clinical relevance.

Missing values were evident in less than 2% of the cases across the chosen variables. Given the sample size of 44,610, they could have been ignored. However, we chose to impute the missing values conservatively in order to have the full sample included in the regression analysis. This means that if a student did not answer a certain item, the item was given the zero or "no" value; for example, if the item for parental separation was not answered, it was counted as "no" for parental separation events. This handling was used for the variables parental warmth and control, parental separation events, cultural communication in the family, number of (delinquent) friends, smoking parents, neighborhood cohesion and safety, living on welfare, violence level in school, willingness of teachers to intervene, aggressive behavior of teachers, volunteer activities, school commitment, social integration in school, planned school leaving certificate, hedonistic reasons for truancy, ADHD, risk-taking behavior, self-esteem, mental well-being, and mandatory repetition of school year. Only items for which social desirability could have been a reason for the missing value - because the item asked, for example, for something that was inconsistent with conventional norms - were missing values imputed with the mean value of the students who answered the item. This worked only when a metric variable was evident. This latter imputation method concerns the variables deviant/assimilated behavior in one's own group of friends, social desirability, average school grades, and school anxiety.

## Results

The prevalence of binge drinking in the sample was 52.3%. The percentage of male adolescents who engaged in binge drinking in the last 30 days was 56.9%, and the percentage of females was 47.5%. The prevalence of dinge drinking in this sample according to migration background and according to urban and rural living place has been published elsewhere [4].

The binary logistic regression analysis resulted in a significant model (*p *< .001) with an explained variance (R^2^) of 27.8% (Chi^2 ^(29) = 10407.5). Using the chosen predictors, the model was able to correctly classify 70.2% of the adolescents as engaging in binge drinking or not. Table 3 shows which predictors had a significant association with binge drinking. They were (independent of direction and strength of association): parental separation, cultural communication in the family, number of friends, deviant behavior in the student's own group of friends, smoking parents, neighborhood cohesion, neighborhood safety, welfare status, willingness of teachers to intervene during violent conflicts, aggressive behavior of teachers, engagement in non-profit volunteer activities, religiosity, school commitment, social integration in school, absenteeism, risk-taking behavior, school grades, suicidal thoughts, and mandatory repetition of a school year. Variables of marginal significance were self-esteem, school anxiety (both p = .001), and mental well-being/mood (p = .004).

**Table 3:** Results of the multiple binary logistic regression analysis with Binge Drinking as dependent variable

Variables*	Regression Co-efficient Beta (β)	Standard Error	Wald	p-value	Odds Ratio	95% Confidence Interval for OR	
						
						Lower Value	Upper Value
Parental Acknowledgment of success	.012	.005	5.536	.019	1.012	1.002	1.023

Parental warmth in childhood	.002	.001	3.571	.059	1.002	1.000	1.004

Parental separation^#^	.129	.025	27.173	**<.001**	1.137	1.084	1.194

Cultural communication in the family	-.035	.004	68.794	**<.001**	.966	.958	.974

Number of friends	.067	.002	1134.215	**<.001**	1.069	1.065	1.073

Deviant behavior in one's group of friends	.099	.006	264.020	**<.001**	1.104	1.091	1.117

Assimilated behavior in one's group of friends	-.010	.006	2.299	.129	.990	.978	1.003

Smoking parents^#^	.080	.023	12.574	**<.001**	1.084	1.037	1.133

Neighborhood cohesion	.026	.003	69.966	**<.001**	1.026	1.020	1.033

Neighborhood safety	.044	.005	95.539	**<.001**	1.045	1.036	1.054

Living on welfare^#^	-.446	.040	126.881	**<.001**	.640	.592	.692

Violence level in school	-.013	.008	2.595	.107	.987	.972	1.003

Willingness of teachers to intervene during violent conflicts	.039	.007	28.532	**<.001**	1.040	1.025	1.055

Violence at school: aggressive behavior of teachers	.231	.023	98.496	**<.001**	1.260	1.204	1.319

Voluntary non-profit activities	.072	.013	29.762	**<.001**	1.074	1.047	1.102

Religiosity	-.292	.010	801.239	**<.001**	.747	.732	.762

School commitment	-.096	.008	153.245	**<.001**	.909	.895	.923

Social integration in school	.145	.008	296.640	**<.001**	1.156	1.137	1.175

Social desirability/Conventional values	-.002	.012	.039	.843	.998	.975	1.021

Planned school leaving certificate	.013	.015	.727	.394	1.013	.983	1.044

Absenteeism/Truancy^#^	.323	.023	188.998	**<.001**	1.381	1.319	1.446

ADHD^#^	-.003	.043	.004	.951	.997	.916	1.086

Risk-taking behavior	.109	.004	737.495	**<.001**	1.115	1.106	1.124

School grades (average)^§^	.170	.016	108.786	**<.001**	1.185	1.148	1.224

Self-esteem	-.012	.004	10.624	.001	.988	.980	.995

Mental well-being/mood	-.013	.004	8.097	.004	.987	.979	.996

School anxiety	-.012	.004	11.787	.001	.988	.981	.995

Suicidal thoughts	.264	.014	378.473	**<.001**	1.303	1.268	1.338

Mandatory repetition of school year^#^	.288	.027	111.281	**<.001**	1.333	1.264	1.407

Constant	-3.449	.144	577.190	<.001	.032		

*excluded because of multicollinearity: parental control/supervision in adolescence; number of delinquent friends; hedonistic reasons for absenteeism/truancy

^# ^Variable coding for dichotomous variables: 0 = no; 1 = yes

^§ ^Grades in the German school system: 1 = "very good" through 6 = "not sufficient"

**Figure 2:**
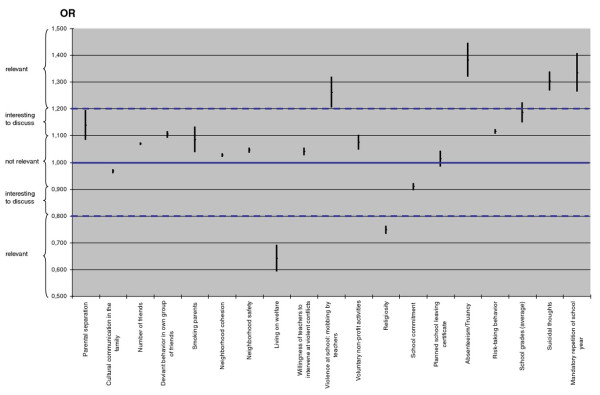
**Odds Ratios including Confidence Intervals of significant predictors (*p *< .001)**.

According to the analysis, there were two clinically relevant protective factors that were identified for binge drinking (Figure 2). First, for a family on welfare, the risk of engaging in binge drinking for the adolescent was about 1/3 lower. Second, if a student rated religion for himself as something personally important, then the risk of engaging in binge drinking was about 1/4 lower. Even though far more risk factors than protective factors were identified, the four most important and relevant risk factors were (Figure 2): 1) Absenteeism/Truancy with about a 1/3 higher risk of engaging in binge drinking if an adolescent had at least once missed a whole day of school without excuse in comparison to students who had never been absent for a whole day without reason or excuse. 2) Mandatory repetition of a school year, which can be interpreted as weak academic skills, and implies an app. 1/3 higher risk of binge drinking in comparison to students who achieved the academic goals of a given school year. 3) Suicidal thoughts, such that students who more often had suicidal thoughts had about a 1/3 higher risk of engaging in binge drinking in comparison to adolescents who never or rarely thought of suicide. Suicidal thoughts can be interpreted as poor coping skills in stressful life situations. 4) The fourth most important risk factor for binge drinking was violence at school, in particular, so-called aggressive behavior of teachers, indicating that the student had experienced a negative situation including verbal violence with a teacher in the last 6 months. For those students, the risk for engaging in binge drinking was about 1/4 higher than for students who had not had any such experience.

Besides the six most relevant influence factors named above, there were five more factors that were flagged as interesting to discuss (Figure 2): I) school grades on average (the worse they were) were found to be a risk factor (app. 19% increased risk) for binge drinking, a finding that is in line with the significant result of mandatory repetition of a school year because of academic failure. II) The degree of social integration in school was found to be a risk factor for binge drinking with an increased risk of about 16% when the integration score was higher. III) Parental separation/divorce was also found to be a risk factor, increasing the risk for binge drinking app. 14% in comparison to students whose parents were not separated/divorced. IV) Furthermore, the analysis showed that when the student himself was prone to risk-taking behavior due to personality, the probability for binge drinking was higher (about 12%).V) Last, if the student's group of friends usually participated in deviant behavior, the risk for binge drinking was also increased (app. 10%).

As sensitivity analysis the whole regression was carried out with the non-imputed data. Results are very similar. They can be found in an additional table (see Additional file 1: Table S1). Furthermore the analysis was carried out separately for boys and girls. The predictors of binge drinking for boys and girls of the 9th grade are mostly identical. There are only four differences out of 29 variables in the analysis compared to the results for all adolescents. For girls are „Assimilated behavior in one's group of friends", „Self-esteem" und „Mental well-being/mood" significant predictors, but not so for male 9th graders. Instead „Willingness of teachers to intervene during violent conflicts" is relevant for male adolescents but not for females (see Additional file 1: Table S2 and Additional file 1: Table S3).

## Discussion

The aim of the study was to analyze ultimate and distal predictors for binge drinking in a representative sample of German adolescents. As a result, two significant and clinically relevant protective factors and four significant and clinically relevant risk factors for binge drinking were identified. Including the marginally relevant and statistically significant predictors (5), the number of risk factors increased to a total of nine.

Most of the findings of this work are in the expected direction and in accordance with the literature for substance use in general. Some of the findings are really new and have not been discussed in other studies so far. The following section discusses the state-of-art knowledge concerning the eleven most relevant risk and protective factors for binge drinking in comparison to the results of our study.

### Suicidal thoughts

In contrast to anxiety, which was not relevantly or not positively associated with binge drinking in our study and in previous studies [41], the presence of depression and emotional problems are known to be positively associated with binge drinking [42] as we discovered also in our study, in which the presence and frequency of suicidal thoughts were strong predictors of binge drinking. Studies that explicitly included suicidal thoughts as predictors of substance use support our results also, even though they were not specifically aimed at binge drinking [43,44].

### School grades & mandatory repetition of school year

Concerning the factors associated with school and academic achievement, the literature shows a clear picture. As in our study, binge drinking has been associated with academic failure (worse school grades and mandatory repetition of a school year) [45,46], which could be the consequence of memory and neurocognitive deficits on the one hand, which have shown to be side effects and direct consequences of binge drinking [47-50]. On the other hand, binge drinking can also be a coping mechanism for dealing with academic failure in comparison to other peers [51,52].

### Welfare status

Other predictors were significant but still surprising. For example, the finding that living on welfare is rather a protective factor for binge drinking even though it is known that living on welfare or poverty in general is a risk factor for many other health factors such as obesity, diabetes, stress, or kidney disease [53-57]. However, comparable to our study, Bellis et al. [58] found in their sample of 11,000 students in England in the same age group as investigated here that children with greater expendable incomes report more unsupervised, frequent, and heavy drinking. The same was found for Spanish adolescents [59]. Also, another study showed that early alcohol use by adolescents is more frequent in higher-income households [60]. The most obvious explanation for this effect may be that binge drinking causes financial expenditures that can rarely be afforded by teenagers with a smaller budget.

### Religiosity

There have been three studies identifying religiosity or spirituality as a protective factor for binge drinking [61-63] as our data also suggest. However, they were all carried out in the U.S. where religion plays a far greater role in daily life than in Germany, especially for younger people. They also showed that a protective effect is evident only for heterosexual adolescents [62] and when religion reaches a certain degree of personal importance in the life of the individual student.

### Social integration

The thesis that binge drinking is a social act or even constitutes a spare-time activity is supported by the finding that social integration and number of friends is also positively associated with engaging in binge drinking in our study, suggesting that heavy alcohol consumption is something that students do together as a collective [see also [64,65] and that binge drinking does not represent a consumption pattern of boredom in Germany as found in England [58]. Our results are supported by a recent study on 26,000 Spanish adolescents for whom social integration in a group of friends who frequently go out and get drunk is predictive of habitual excessive drinking [59].

### Risk-taking behavior

Concerning intrapersonal factors, Wechsler et al. [65] found, as we did, that risk-taking behavior is positively associated with binge drinking in U.S. college students. Also, Neumark et al. [66] showed that different risky behaviors are associated with binge drinking in adolescents from different cultural/religious backgrounds.

### Parental separation

The risk of adolescent binge drinking due to parental separation has not been explicitly shown so far. Only in adults a person's own divorce has been shown to be a significant risk factor for binge drinking [67,68]. As we have shown, a risk behavior such as binge drinking in adolescents can already be a consequence of experiences with parental divorce and therefore of a change or even loss in the own family - and social support system. Thus, binge drinking after parental separation could be interpreted as a coping mechanism for this critical life event.

### Deviant behavior in one's own group of friends & truancy

Since there seem to be associations between delinquent behavior, deviant behavior in a person's own peer group, and truancy/absenteeism, these concepts and their roles in the literature are discussed together. These variables are among the strongest predictors of binge drinking in our study. Also, other authors have found that engaging in deviant behaviors (together with antisocial peers [69]) foretells binge drinking in adolescents [51,52,69] and is additionally often associated with truancy and "hanging around during the day" [51,59,66,70]. Remarkably, the association of binge drinking with truancy has been shown in different European countries and provides support for our results in Germany. It is also known from tobacco research that the number of friends and their substance-related behavior, even if it is against the social/legal norms, have strong impacts on the consumption behavior of the adolescent himself [71].

### Violence at school: aggressive behavior of teachers

The relatively strong impact of violence at school, in particular, in the form of aggressive behavior of teachers - be it verbally or other - was rather surprising. There have been no published studies known to the authors thus far that have investigated this construct and its role in consumption behavior. Thus, this finding is new and adds an additional piece of knowledge to understanding excessive consumption behaviors in adolescents for the scientific community. There are different possible ways to interpret this result: 1. Adolescence as a developmental phase is characterized by a high vulnerability for being hurt. In the case of low frustration tolerance, a slight or offensive behavior by someone higher in the hierarchy could result in problematic behavior such as binge drinking, through which the person regains his/her strength and reputation in the peer group (compensation mechanism). 2. It is also plausible that teachers know which adolescents engage more in deviant behavior (like truancy) or problematic risky behavior (like binge drinking) than others because of the students' academic achievements. It could be that either consciously or unconsciously, the teachers react more aggressively toward those adolescents.

Trying to integrate the results into the theory of triadic influence suggested by Petraitis et al., who developed the model for different substances ("Illicit Substance Use"), it can be seen that almost all intrapersonal distal factors are important for predicting binge drinking, but not all ultimate intrapersonal factors are. It has to be taken into account that only part of the suggested ultimate intrapersonal factors could be operationalized. Concerning the attitudinal/environmental factors, all ultimate factors named by Petraitis showed predictive power also for binge drinking, even though the policy factors were not operationalized since they are the same for all analyzed adolescents in the sample because of the federal German alcohol policy. The distal attitudinal/environmental factors named by Petraitis could not all be proven to be predictive of binge drinking: Whereas commitment to school and religion, as well as hedonic values and tolerance of deviance showed statistical significance, the commitment to conventional values did not serve as a predictor, and engagement in non-profit volunteer activities showed a different direction of association, as expected. For the social/interpersonal distal factors, the model of Petraitis was basically supported for binge drinking; for the ultimate factors, however, only part of the constructs are relevant for binge drinking. Whereas home strain, parental separation, and unconventional values and behaviors of parents were statistically significantly associated with binge drinking, factors such as parental warmth or acknowledgment of success by parents did not have significant predictive power. It has to be taken into account that the chosen and analyzed items are the best approximation to the theoretical model of Petraitis that was possible for us. The study was not designed in the first place to prove this specific model, and therefore not all influence factors could be operationalized. However, out of every distal and ultimate category of the model, several variables were available to operationalize the constructs.

It is true that the cross-sectional study presented is based on a representative sample, however, there are limitations concerning the composition of variables and the study design. Variables asking for adolescents' beliefs referring to Petraitis et al. proximal influence factors were not assessed. Therefore the indisputable impact of these variables on binge drinking cannot be described in the context of our variables. Furthermore the study is limited on 9^th ^graders. Thus we cannot compare our results with younger or older adolescents. Our survey is a cross-sectional study, for this reason we have no information on changes in binge drinking behaviors and their influencing factors.

The prevalence of binge drinking in the surveyed sample of 9^th ^graders lies, on the one hand, in the expected range (20% to 60%) that is reported by other large studies [12,13,15]. On the other hand, a representative investigation in Germany of 12-to-17-year-olds reported much lower levels of Binge Drinking just as was found by a review in the US investigating Binge Drinking in 12-to-20-year-olds [5,14]. An explanation could be that in our study, only 9^th ^graders with a mean age of 15 years were included, and the standard deviation of the currently surveyed age group was a lot smaller (SD = 0.7). Obviously, this age group is a lot more engaged in substance consumption activities than 12-year-olds. Still, the percentages of 56.9% for males and 47.5% for females are likely to be valid and probably not overestimated. The analyses of the study were based on a large representative sample of adolescents, suggesting validity of the data. However, it has to be taken into account that the adolescents who refused to participate probably engage at least to the same percentage as their participating colleagues in alcohol consumption, maybe even more, since they were unwilling to disclose this behavior. It could therefore even be possible that the number of consumers or specifically binge drinkers is slightly underestimated. The fact that the prevalence of binge brinking is higher in boys than in girls was also found in other studies e. g. [72]. This representative sample supports that difference in consumption patterns.

Whereas some of the influence factors for binge drinking are not surprising since they are known predictors of substance abuse in general (e.g., risk-taking behavior), there are two points that could be targeted in interventions that should not focus on adolescents alone: 1. training teachers in positive, reassuring behavior and constructive criticism which has also been proofed as effective pedagogic strategy in intervention studies (for example [73]) and 2. a focus on high risk students either because they lack coping strategies when in negative mood or because of their low academic achievements and absenteeism from school. An interesting fact is also the protective value of religiosity, which so far has not been the focus of prevention strategies especially not in a country like Germany.

Further research on Binge Drinking in adolescents should try to include all facets of Petraitis' model as predictors or be theoretically based on alternative models. Furthermore, research should focus on adolescents of different age groups and aim for longitudinal studies.

## Conclusions

In conclusion, new insights of predictors of binge drinking especially for the European context could be generated. Based on a theoretical model we could show that the two most influential protective factors against Binge Drinking were low economic status and importance of religion and that school absenteeism/truancy, academic failure, suicidal thoughts, and aggressive behavior of teachers are among the most influential risk factors for binge drinking.

## Competing interests

The authors declare that they have no competing interests.

## Authors' contributions

CD carried out data analysis and drafted the manuscript. DB worked out the Methods section. EG contributed and wrote parts of the Discussion and Conclusions. TH and SB organized the study and revised the manuscript. CP was head of the data collection team and helped with the data transfer. All authors read and approved the final manuscript.

## Pre-publication history

The pre-publication history for this paper can be accessed here:

http://www.biomedcentral.com/1471-2458/12/263/prepub

## Supplementary Material

Additional file 1 **Table S1**. Results of the multiple binary logistic regression analysis with non-imputed data (n = 34,116; 76.5% of the sample) with Binge Drinking as dependent variable (R^2 ^= 29.0%; Chi^2 ^= 8367.8 *p *< .001; correctly classified 70.7%). **Table S2**. Results of the multiple binary logistic regression analysis for male adolescents with Binge Drinking as dependent variable (R^2 ^= 25.4%; Chi^2 ^= 4749.2 *p *< .001; correctly classified 70.2%). **Table S3**. Results of the multiple binary logistic regression analysis for female adolescents with Binge Drinking as dependent variable (R^2 ^= 29.5%; Chi^2 ^= 5374.0 *p *< .001; correctly classified 70.7%). Click here for file
