# Supplementary material for: Predictors of binge drinking in adolescents: ultimate and distal factors - a representative study
Source: BMC Public Health. 2012 Apr 2;12:263. doi: 10.1186/1471-2458-12-263 (PMC3378431; doi:10.1186/1471-2458-12-263)
Supplement: Additional file 1 — Table S1. Results of the multiple binary logistic regression analysis with non-imputed data (n = 34,116; 76.5% of the sample) with Binge Drinking as dependent variable (R2 = 29.0%; Chi2 = 8367.8 p < .001; correctly classified 70.7%). Table S2. Results of the multiple binary logistic regression analysis for male adolescents with Binge Drinking as dependent variable (R2 = 25.4%; Chi2 = 4749.2 p < .001; correctly classified 70.2%). Table S3. Results of the multiple binary logistic regression analysis for female adolescents with Binge Drinking as dependent variable (R2 = 29.5%; Chi2 = 5374.0 p < .001; correctly classified 70.7%). [file 1471-2458-12-263-S1.DOC]

**Additional Table 1: Results of the multiple binary logistic regression analysis with non-imputed data (n = 34,116; 76.5% of the sample) with Binge Drinking as dependent variable (R² = 29.0%; Chi² = 8367.8 p < .001; correctly classified 70.7%)**

| Variables* | Regression Co-efficient Beta (β) | Standard  Error | Wald | p-value | Odds Ratio | 95% Confidence Interval for OR | |
| --- | --- | --- | --- | --- | --- | --- | --- |
|  |  |  |  |  |  | Lower Value | Upper Value |
| Parental Acknowledgment of success | .012 | .006 | 3.718 | .054 | 1.012 | 1.000 | 1.024 |
| Parental warmth in childhood | -.001 | .001 | .201 | .654 | .999 | .997 | 1.002 |
| Parental separation^#^ | .126 | .028 | 19.888 | **<.001** | 1.135 | 1.073 | 1.200 |
| Cultural communication in the family | -.033 | .005 | 47.237 | **<.001** | .967 | .958 | .976 |
| Number of friends | .076 | .002 | 1036.641 | **<.001** | 1.079 | 1.074 | 1.084 |
| Deviant behavior in one’s group of friends | .111 | .007 | 223.869 | **<.001** | 1.117 | 1.101 | 1.133 |
| Assimilated behavior in one’s group of friends | -.013 | .007 | 3.010 | .083 | .987 | .973 | 1.002 |
| Smoking parents^#^ | .116 | .034 | 11.658 | .001 | 1.123 | 1.051 | 1.201 |
| Neighborhood cohesion | .032 | .004 | 73.841 | **<.001** | 1.033 | 1.025 | 1.041 |
| Neighborhood safety | .044 | .006 | 52.294 | **<.001** | 1.045 | 1.033 | 1.058 |
| Living on welfare^#^ | -.433 | .045 | 93.551 | **<.001** | .648 | .594 | .708 |
| Violence level in school | -.015 | .010 | 2.203 | .138 | .986 | .967 | 1.005 |
| Willingness of teachers to intervene during violent conflicts | .024 | .009 | 7.214 | .007 | 1.024 | 1.007 | 1.043 |
| Violence at school: aggressive behavior of teachers | .201 | .027 | 56.291 | **<.001** | 1.223 | 1.160 | 1.288 |
| Voluntary non-profit activities | .094 | .015 | 38.789 | **<.001** | 1.099 | 1.067 | 1.132 |
| Religiosity | -.336 | .012 | 801.987 | **<.001** | .715 | .698 | .732 |
| School commitment | -.102 | .009 | 124.813 | **<.001** | .903 | .887 | .920 |
| Social integration in school | .140 | .010 | 182.491 | **<.001** | 1.150 | 1.127 | 1.174 |
| Social desirability/Conventional values | -.013 | .013 | .975 | .324 | .987 | .961 | 1.013 |
| Planned school leaving certificate | -.028 | .018 | 2.387 | .122 | .972 | .937 | 1.008 |
| Absenteeism/Truancy ^#^ | .346 | .027 | 159.483 | **<.001** | 1.413 | 1.340 | 1.491 |
| ADHD ^#^ | .049 | .051 | .947 | .330 | 1.050 | .951 | 1.160 |
| Risk-taking behavior | .113 | .005 | 573.652 | **<.001** | 1.120 | 1.109 | 1.130 |
| School grades (average)^§^ | .177 | .019 | 87.093 | **<.001** | 1.194 | 1.150 | 1.239 |
| Self-esteem | -.016 | .005 | 12.113 | .001 | .984 | .975 | .993 |
| Mental well-being/mood | -.011 | .005 | 4.108 | .043 | .989 | .978 | 1.000 |
| School anxiety | -.008 | .004 | 3.278 | .070 | .992 | .984 | 1.001 |
| Suicidal thoughts | .146 | .016 | 80.914 | **<.001** | 1.157 | 1.121 | 1.194 |
| Mandatory repetition of school year ^#^ | .278 | .032 | 75.570 | **<.001** | 1.320 | 1.240 | 1.406 |
| Constant | -2.992 | .198 | 227.647 | <**.001** | .050 |  |  |

*excluded because of multicollinearity: parental control/supervision in adolescence; number of delinquent friends; hedonistic reasons for absenteeism/truancy

^#^ Variable coding for dichotomous variables: 0 = no; 1 = yes

^§^ Grades in the German school system: 1 = “very good” through 6 = “not sufficient”

**Additional Table 2: Results of the multiple binary logistic regression analysis for male adolescents with Binge Drinking as dependent variable (R² = 25.4%; Chi² = 4749.2 p < .001; correctly classified 70.2%)**

| Variables* | Regression Co-efficient Beta (β) | Standard  Error | Wald | p-value | Odds Ratio | 95% Confidence Interval for OR | |
| --- | --- | --- | --- | --- | --- | --- | --- |
|  |  |  |  |  |  | Lower Value | Upper Value |
| Parental Acknowledgment of success | .018 | .007 | 6.510 | .011 | 1.019 | 1.004 | 1.033 |
| Parental warmth in childhood | .004 | .002 | 8.169 | .004 | 1.004 | 1.001 | 1.007 |
| Parental separation^#^ | .115 | .035 | 10.984 | .001 | 1.122 | 1.048 | 1.201 |
| Cultural communication in the family | -.023 | .006 | 15.932 | **<.001** | .977 | .966 | .988 |
| Number of friends | .058 | .003 | 451.587 | **<.001** | 1.060 | 1.054 | 1.065 |
| Deviant behavior in one’s group of friends | .076 | .007 | 108.830 | **<.001** | 1.079 | 1.063 | 1.094 |
| Assimilated behavior in one’s group of friends | .013 | .009 | 2.395 | .122 | 1.013 | .996 | 1.031 |
| Smoking parents^#^ | .076 | .032 | 5.691 | .017 | 1.079 | 1.014 | 1.148 |
| Neighborhood cohesion | .025 | .004 | 33.052 | **<.001** | 1.025 | 1.016 | 1.034 |
| Neighborhood safety | .036 | .006 | 37.159 | **<.001** | 1.037 | 1.025 | 1.049 |
| Living on welfare^#^ | -.580 | .058 | 99.234 | **<.001** | .560 | .499 | .627 |
| Violence level in school | -.019 | .011 | 3.140 | .076 | .981 | .961 | 1.002 |
| Willingness of teachers to intervene during violent conflicts | .055 | .010 | 30.758 | <**.001** | 1.056 | 1.036 | 1.077 |
| Violence at school: aggressive behavior of teachers | .215 | .033 | 43.404 | **<.001** | 1.240 | 1.163 | 1.322 |
| Voluntary non-profit activities | .060 | .019 | 9.838 | .002 | 1.062 | 1.023 | 1.102 |
| Religiosity | -.253 | .014 | 310.266 | **<.001** | .777 | .755 | .799 |
| School commitment | -.094 | .010 | 80.564 | **<.001** | .910 | .892 | .929 |
| Social integration in school | .156 | .012 | 178.679 | **<.001** | 1.168 | 1.142 | 1.195 |
| Social desirability/Conventional values | .006 | .016 | .164 | .686 | 1.006 | .976 | 1.037 |
| Planned school leaving certificate | .008 | .021 | .157 | .692 | 1.008 | .968 | 1.050 |
| Absenteeism/Truancy ^#^ | .275 | .033 | 69.901 | **<.001** | 1.317 | 1.235 | 1.405 |
| ADHD ^#^ | .061 | .054 | 1.282 | .258 | 1.063 | .956 | 1.182 |
| Risk-taking behavior | .109 | .005 | 414.482 | **<.001** | 1.116 | 1.104 | 1.127 |
| School grades (average)^§^ | .178 | .023 | 60.895 | **<.001** | 1.195 | 1.143 | 1.249 |
| Self-esteem | -.009 | .005 | 2.792 | .095 | .991 | .981 | 1.001 |
| Mental well-being/mood | .005 | .007 | .637 | .425 | 1.005 | .992 | 1.018 |
| School anxiety | -.015 | .005 | 7.828 | .005 | .985 | .975 | .996 |
| Suicidal thoughts | .256 | .022 | 137.250 | **<.001** | 1.291 | 1.237 | 1.348 |
| Mandatory repetition of school year ^#^ | .306 | .037 | 68.261 | **<.001** | 1.358 | 1.263 | 1.461 |
| Constant | -3.766 | .192 | 386.297 | <**.001** | .023 |  |  |

*excluded because of multicollinearity: parental control/supervision in adolescence; number of delinquent friends; hedonistic reasons for absenteeism/truancy

^#^ Variable coding for dichotomous variables: 0 = no; 1 = yes

^§^ Grades in the German school system: 1 = “very good” through 6 = “not sufficient”

**Additional Table 3: Results of the multiple binary logistic regression analysis for female adolescents with Binge Drinking as dependent variable (R² = 29.5%; Chi² = 5374.0 p < .001; correctly classified 70.7%)**

| Variables* | Regression Co-efficient Beta (β) | Standard  Error | Wald | p-value | Odds Ratio | 95% Confidence Interval for OR | |
| --- | --- | --- | --- | --- | --- | --- | --- |
|  |  |  |  |  |  | Lower Value | Upper Value |
| Parental Acknowledgment of success | .009 | .008 | 1.293 | .256 | 1.009 | .994 | 1.024 |
| Parental warmth in childhood | .000 | .002 | .023 | .880 | 1.000 | .997 | 1.003 |
| Parental separation^#^ | .149 | .036 | 17.395 | **<.001** | 1.161 | 1.082 | 1.245 |
| Cultural communication in the family | -.049 | .006 | 62.078 | **<.001** | .952 | .940 | .964 |
| Number of friends | .079 | .003 | 700.949 | **<.001** | 1.082 | 1.076 | 1.088 |
| Deviant behavior in one’s group of friends | .157 | .012 | 183.870 | **<.001** | 1.171 | 1.144 | 1.198 |
| Assimilated behavior in one’s group of friends | -.037 | .010 | 13.938 | <**.001** | .964 | .946 | .983 |
| Smoking parents^#^ | .075 | .033 | 5.205 | .023 | 1.078 | 1.011 | 1.151 |
| Neighborhood cohesion | .027 | .005 | 35.173 | **<.001** | 1.028 | 1.019 | 1.037 |
| Neighborhood safety | .049 | .007 | 46.625 | **<.001** | 1.051 | 1.036 | 1.066 |
| Living on welfare^#^ | -.321 | .055 | 34.116 | **<.001** | .726 | .652 | .808 |
| Violence level in school | -.013 | .012 | 1.113 | .291 | .987 | .965 | 1.011 |
| Willingness of teachers to intervene during violent conflicts | .017 | .011 | 2.391 | .122 | 1.017 | .995 | 1.039 |
| Violence at school: aggressive behavior of teachers | .237 | .034 | 48.742 | **<.001** | 1.268 | 1.186 | 1.355 |
| Voluntary non-profit activities | .089 | .019 | 23.021 | **<.001** | 1.093 | 1.054 | 1.133 |
| Religiosity | -.327 | .015 | 464.552 | **<.001** | .721 | .700 | .743 |
| School commitment | -.093 | .012 | 62.786 | **<.001** | .911 | .891 | .933 |
| Social integration in school | .130 | .012 | 110.348 | **<.001** | 1.139 | 1.112 | 1.167 |
| Social desirability/Conventional values | -.006 | .018 | .126 | .722 | .994 | .959 | 1.029 |
| Planned school leaving certificate | .020 | .023 | .744 | .388 | 1.020 | .975 | 1.066 |
| Absenteeism/Truancy ^#^ | .376 | .034 | 121.090 | **<.001** | 1.457 | 1.363 | 1.558 |
| ADHD ^#^ | -.145 | .074 | 3.840 | .050 | .865 | .748 | 1.000 |
| Risk-taking behavior | .100 | .006 | 254.056 | **<.001** | 1.106 | 1.092 | 1.119 |
| School grades (average)^§^ | .148 | .024 | 38.631 | **<.001** | 1.159 | 1.107 | 1.215 |
| Self-esteem | -.021 | .006 | 13.041 | <**.001** | .979 | .968 | .991 |
| Mental well-being/mood | -.030 | .006 | 22.525 | **<.001** | .970 | .958 | .982 |
| School anxiety | -.006 | .005 | 1.318 | .251 | .994 | .984 | 1.004 |
| Suicidal thoughts | .265 | .019 | 203.043 | **<.001** | 1.304 | 1.257 | 1.352 |
| Mandatory repetition of school year ^#^ | .270 | .041 | 43.140 | **<.001** | 1.310 | 1.209 | 1.420 |
| Constant | -2.945 | .223 | 174.252 | <**.001** | .053 |  |  |

*excluded because of multicollinearity: parental control/supervision in adolescence; number of delinquent friends; hedonistic reasons for absenteeism/truancy

^#^ Variable coding for dichotomous variables: 0 = no; 1 = yes

^§^ Grades in the German school system: 1 = “very good” through 6 = “not sufficient”
